# A Scoping Review on Bone Mass Status in Individuals with Transfemoral Amputation

**DOI:** 10.1097/JPO.0000000000000635

**Published:** 2026-05-19

**Authors:** Jose L. Zavaleta-Ruiz, Matthew J. Major, Pankaj Pankaj

**Affiliations:** JOSE L. ZAVALETA-RUIZ, PhD, MPO and PANKAJ PANKAJ, PhD, are affiliated with School of Engineering, The University of Edinburgh, Edinburgh, UK.; MATTHEW J. MAJOR, PhD, is affiliated with Departments of Physical Medicine & Rehabilitation, Biomedical Engineering, and Mechanical Engineering, Northwestern University, Jesse Brown VA Medical Center, Chicago, Illinois.

**Keywords:** bone mineral density, osteopenia, prosthesis suspension, transfemoral amputation

## Abstract

**Background:**

Deterioration of bone mineral density (BMD) in the residual femur after a transfemoral amputation (TFA) has been reported over the past five decades, with various potential explanations described in the literature. BMD loss is a critical risk factor of fracture and hence an important clinical concern for prosthesis users.

**Objectives:**

This scoping review audited in vivo imaging studies that focused on BMD in individuals with TFA, with the aim to synthesize the status of knowledge on this topic, report on the mechanisms related to BMD status as hypothesized in the reviewed literature, and review BMD outcomes separately for prosthetic socket and bone-anchored suspension.

**Methods:**

Three databases (Scopus, PubMed, and Web of Science) were searched on December 1, 2024. Search keywords covered terms that reference bone mineralization (BMD, osteopenia, and osteoporosis) and TFA. Data were extracted for analysis and grouped according to reported prosthesis suspension for analysis.

**Results:**

In total, 244 articles were screened, with 20 ultimately reviewed. Articles were published between 1969 and 2024. Fourteen articles reported the use of socket prosthesis and six used bone-anchored devices. The former group consistently reported BMD loss, while the latter presented maintenance or improvements. The socket prosthesis cohort was younger in age compared to bone anchored (range 24–73 vs. 47–58 years). Proposed reasons for BMD loss included disuse atrophy, bedrest post-surgery, gait asymmetry, and prosthesis influence.

**Conclusions:**

Articles reporting BMD status using socket prosthesis consistently reported demineralization despite interventions, with no consensus on the mechanical cause of BMD loss. Further, the bone-anchored group tended to demonstrate improvements in limb loading symmetry during ambulation and higher levels of bone loading compared to prosthetic socket use.

**Clinical relevance:**

The residual femur in persons with TFA has demonstrated bone demineralization, which appears to be less prevalent in bone-anchored devices compared to socket use. These findings warrant further analysis of the effects of prosthetic socket use on bone demineralization in persons with TFA.

First radiographic evidence of bone deterioration obtained through X-rays in persons with transfemoral amputation (TFA) wearing a prosthesis was reported in 1969 and then in 1978.^1,2^ Subsequent research has confirmed this trend of bone mineral density (BMD) loss using dual-energy X-ray absorptiometry (DEXA).^3,4^ Evidence of this pattern of BMD loss continues to be reported in present time,^5,6^ despite prosthetic technological progress, highlighting the continued risk of osteoporosis in persons with TFA^7^ and the need to address this problem.

Prosthetic efficacy has often been measured by its capacity to yield close to able-bodied performance outcomes in prosthesis users, while offering high levels of perceived comfort.^8^ Among the several components in a conventional prosthesis is the socket that serves as the coupling interface between the residual limb and the artificial limb and has a direct influence on user comfort. The socket has evolved from the wood carved “plug fit” prior to World War II, through the quadrilateral socket post-Vietnam-American war, to today’s commonly employed ischial containment socket (ICS)^9,10^ and the more recent sub-ischial sockets.^11,12^ The simplicity and high functionality of the ICS^13,14^ has made it the standard of care since its inception in the 1980s.^10^ Distinct from socket suspension, the bone-anchored suspension (also known as *osseointegration*) technique, introduced in the early 1990s, involves surgically implanting a prosthesis into the residual bone and was proposed as an alternative suspension method to obviate the need of a socket and its related complications.^15^ The risks of a surgical endoprosthetic intervention include stoma infection at the penetration site and stress shielding (well known in joint replacements) that could encourage implant loosening have prevented this technique from becoming a standard to replace the socket-based prosthesis.^16^ However, successful bone-anchored interventions have reported improved prosthesis control and comfort when sitting^17^ and recently suggested an improvement in retaining BMD.^18^

It is well recognized that bone health is based on the remodeling dogma explained by Wolff’s law,^19^ wherein bone adapts to the loads under which it is placed via the process of mechanotransduction,^20^ and it is regulated by factors such as age, sex, and life styles.^21^ Able-bodied older adults combining weight-bearing/impact loading training have shown BMD improvements.^22,23^ This relationship might suggest that young individuals with a TFA who participate in high-impact activities would demonstrate BMD gains or maintenance. However, this expectation is not supported by the evidence.^5,6,24^ Observed deterioration levels measured via DEXA imply that individuals with TFA are at risk of hip fractures, which is clinically relevant given the increased likelihood of falling in this patient group.^25^ In accordance with mechanotransduction, certain developments in modular prosthetic components (sockets, liners, pylons, feet) that aim to improve user performance and comfort during daily activities and minimize the risk of soft tissue insults^26–33^ may be partially responsible for the ongoing phenomenon of BMD loss in persons with TFA. For instance, elastomeric liners are regularly used as added interface layers to distribute pressure over sensitive areas,^34^ socket designs with ischial containment redirect forces to the pelvis to minimize loading the residuum,^35^ and contemporary modular pylons and feet^36–38^ are able to better manage energy transfer from ground forces to the residual limb and attenuate energy absorbed by the residuum tissue to shield it from potentially harmful stressors.

The consistency of reports of BMD loss in persons with TFA over the past years,^5,6,24^ and recent reports of BMD recovery in this patient group with bone-anchored prosthesis,^18,39^ raises questions regarding the role of prosthesis design in bone demineralization. The aim of this scoping review was to summarize the status of knowledge on this phenomenon, grouped by either prosthetic socket or bone-anchored suspension, by reporting from each study the sample characteristics, bone status, measurement techniques, mechanisms related to bone status, and suggested recommendations to alter it. This review aimed to present the potential multimodal rationales related to the progression of BMD loss and potentially identify gaps in the literature that may warrant further study.

## METHODOLOGY

### SEARCH STRATEGY

This scoping review searched three comprehensive and up-to-date databases for health/allied health, biomedical, and engineering research (Scopus, PubMed, and Web of Science) on December 1, 2024 for relevant articles using the same search query, that cover variations of keywords related to BMD and TFA: (Osteopenia OR Osteoporosis OR Bone mineral density) AND (amput* AND (transfemoral OR above*knee)).

### STUDY SELECTION

A full list of articles resulting from the search keys was collated into a database by title, author, and year. There was no restriction on publication year. Duplicates were removed.

Articles were initially screened by title/abstract for relevance. Initially articles were excluded if published as conference proceedings or not original research (e.g., reviews, editorials, perspectives, opinions). Review articles were retrieved to extract relevant articles for screening and evaluation. The full texts of articles were evaluated only if available in English.

The retrieved full texts of relevant articles were evaluated against the selection criteria. Articles were included if they reported a quantifiable BMD evaluation through medical imaging in persons with TFA at the ipsilateral hip joint (i.e., regardless of comparison with contralateral side or a control group). Articles were excluded if they reported on multiple levels of amputation or generally on persons with nondescript “lower limb amputation” for which data exclusively to TFA could not be extracted and if they discussed endoprosthetics.

### DATA EXTRACTION

The data extracted from each article were inserted into a spreadsheet including prosthesis suspension used (socket type or bone anchored), authors, publication year, TFA sample description (age, sex distribution, activity-based division), amputated side bone status at time of study, proposed cause for bone status, and recommendations to alter bone status. Articles that reported on bone grading scale and imaging technique were also extracted. Finally, articles that described risk factors for BMD status alterations were also extracted.

## RESULTS

**Figure 1.**
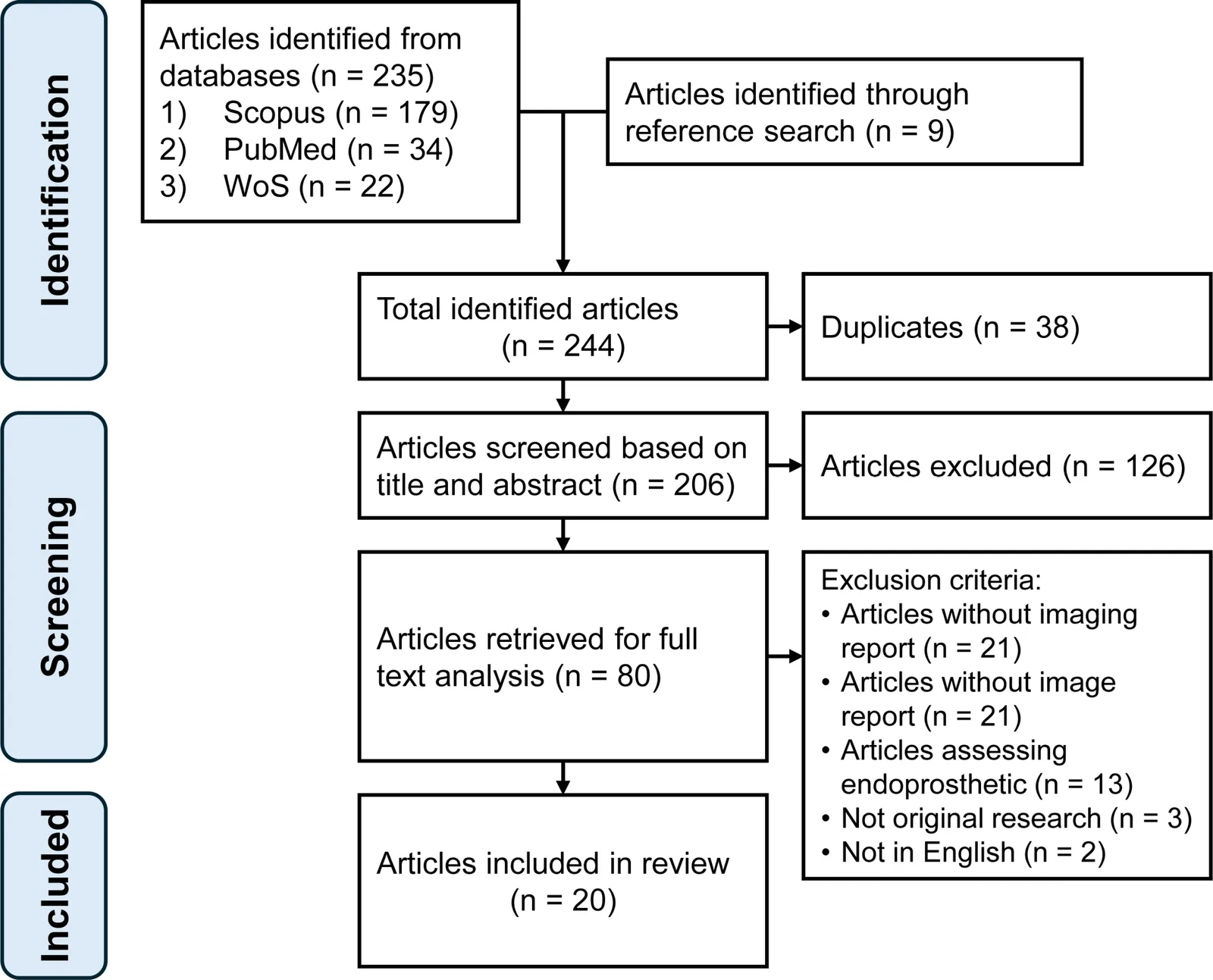
Flowchart displaying the screening and selection process and results. BMD, bone mineral density; TFA, transfemoral amputation.

The initial search criteria yielded 244 articles, with screening and evaluation results displayed in Figure 1. Selection criteria ultimately yielded a total of 20 articles included in this review with BMD reports in individuals with TFA.

The use of socket prosthesis was reported in 14 articles, Table 1. With sample sizes from 3 to 156 individuals. The mean participant age ranged from 24 to 73 years. Further, seven articles reported on cohorts with military affiliation (Veterans or Servicemembers),^4,5,40,44,46,48,49^ and two reported on high-performance athletes.^45,47^ In socket prosthesis users, four causes were listed for the observation of low BMD level, with 10 articles providing one or more as possible causes,^3–5,40,42–44,46,47,49^ including disuse muscular atrophy (n=4), bedrest following the amputation surgery (n=4), altered loading when wearing a prosthetic socket (n=4), and biomechanical deviations imposed by muscle amputation and asymmetric gait (n=4). Articles provided the following recommendations to alter bone status, increase leg loading (n=4), initialize rapid mobilization (n=3), explore socket load transfer (n=2), establish early monitoring and follow-up screening (n=2), nutritional supplementation (n=2), and surgically restoration of muscle fixation following amputation (n=1). However, in 1969 bone deterioration following a TFA was considered imminent, and in 2023 it was described as unlikely to be recovered.

**Table 1.** Articles with analyzed sample using traditional socket prosthesis

First author, year	Sample description	Amputated side bone status	Proposed cause for bone status	Proposed recommendations
Sevastikoglou et al., 1969^1^	28 subjects with uTFA 10 females 18 males Mean age: 52.7 yearso	Mean cortical thinning of 5.1 mm	None shared	TFA bone deterioration is imminent, whilst for TTA is transitional
Benichou and Wirotius, 1982^40^	53 veterans with uTFA Mean age: 32 years	53 subjects with osteoporosis	Disuse atrophy	Initiate mobilization during the first 12 weeks
Rush et al., 1994^3^	16 males with uTFA Mean age: 48 years	13 subjects with over 20% BMD loss and 3 with less	Socket influence	Analyze BMD levels in TTA to determine socket design factors
Jaegers et al., 1995^41^	12 males with uTFA Mean age: 38.1 years	10 subjects with 10%–20% cortical bone atrophy	None shared	Surviving muscle needs fixation to reduce muscle atrophy
Kulkarni et al., 1998^4^	15 male veterans with uTFA Mean age: 73 years	Mean BMD 16% lower than non-amputated limb	Disuse atrophy and gait asymmetry	Increase load placed in amputated leg
Sherk et al., 2008^42^	7 subjects with uTFA 6 males 1 female Mean age: 44.5 years	5 subjects with osteoporosis and 1 with osteopenia	Disuse atrophy and gait (load) asymmetry	Use of immediate postoperative prosthesis
Ramirez et al., 2011^43^	20 subjects with uTFA 3 females 17 males Mean age: 44.6 yearso	18 subjects with 38% bone stiffness decrease	Socket influence	Modify current load transfer of exoprosthesis to replicate normal load state
Flint et al., 2014^44^	156 combatants 58 subjects with TFA Mean age: 24 years	41 TFA limbs with osteopenia	Disuse atrophy and immobilization	Initiate early and aggressive weight-bearing rehabilitation and secure proper calcium and vitamin D levels
Cavedon et al., 2021^45^	11 male athletes with uTFA Mean age: 34.9 years	4 subjects with osteopenia	None shared	Adapt nutrition and training program disability specific
Hoyt et al., 2021^46^	26 male combatants with 13 TFA and 17 TTA Mean age: 26.4 years	5 TFA limbs with osteopenia and 2 with osteoporosis	Socket influence, disuse atrophy and immobilization	Explore use of CT scans for bone health monitoring
Cavedon et al., 2022^47^	10 male athletes with uTFA Mean age: 36.8 years	8 subjects in the osteopenia–osteoporotic range	Immobilization, Socket influence, and gait (loading) asymmetry	Site-specific (hip level) training
Hoyt et al., 2023^5^	128 combatants with 82 TFA or KD 127 males 1 female Mean age: 24.5 years	65 TFA or KD limbs in the osteopenia–osteoporotic range	Immobilization and surgical complications	Return to normal BMD levels will likely never occur
Yasmeen et al., 2024^48^	3 male combatants with 5 TFA Mean age: 30.8 years	5 TFA limbs with osteoporosis	None shared	Early screening for timely diagnosis.
Jin et al., 2024^49^	11 male veterans with uTFA Mean age: 69 years	Mean BMD in the osteopenia–osteoporosis range	Gait asymmetry	Improve gait loading patterns post amputation

BMD, bone mineral density; KD, knee disarticulation; TFA, transfemoral amputation; TTA, transtibial amputation; uTFA, unilateral TFA.

Table 2 presents the six articles reporting use of bone-anchored prosthesis. Mean participant age was 47–58 years, with a sample size of 5–24 individuals. Only one study reported on a cohort with Veterans affiliation.^39^ All articles offered one or more possible causes for observed low BMD levels prior to surgical intervention, all of which overlapped with the socket prosthesis studies. Moreover, in the bone-anchored prosthesis group, observations of BMD gains/maintenance were attributed to improved gait loading symmetry (n=4), skeletal loading restoration due to elimination of the socket interference (n=3), and renewed muscular activity (n=2). All articles suggest improvement in bone status with longer observation times.

**Table 2.** Articles with analyzed sample using bone-anchored prosthesis

First author, year	Sample description	Amputated side bone status (pre-op)	Proposed cause for bone status	Proposed recommendations
Haket et al., 2017^50^	26 subjects with uTFA and 1 bilateral TFA 21 males 5 females Mean age: 48 years	24 subjects with disuse osteoporosis	Disuse atrophy, and surgical technique depending	Osteoporosis was not reversed after 2 years. Longer follow-up is advised
Hansen et al., 2019^51^	19 subjects with uTFA 12 males 7 females Mean age: 49 years	BMD 36% lower than non-amputated leg	Disuse atrophy, and gait loading asymmetry	Calcium and vitamin D supplements. Implant allowed return at baseline values at 30-month follow-up
Thomson et al. 2019^52^	33 subjects with uTFA 22 males 11 females Mean Age: 51.1 years	33 subjects with osteoporosis	Gait loading asymmetry	Implants stimulate bone remodeling in the femoral neck with significant BMD increases in all subjects
Cano et al., 2020^53^	13 uTFA 10 males 3 females Mean age: 46.9 years	13 subjects with osteopenia pre and 14-month post-op	Gait asymmetry, poor socket fit, stress shielding, and immobilization	3% of BMD increase (no significant). Further improvements in BMD are expected in a longer follow-up period
Sinclair et al., 2022^39^	8 male veterans with uTFA Mean age: 48.8 years	No reported status. Mean BMD = 0.84 ± 0.21	Absence of direct bone loading	4% of BMD increase (no significant) after 24 months. Bone implant demonstrated benefits over socket prostheses
Hoellwarth et al., 2023^18^	5 subjects with uTFA 4 males 1 female Mean age: 57.8 years	5 subjects with osteoporosis.	Altered bone loading, and gait unloading asymmetry	5-year follow-up reported a significant 35% increase. Bone implant promotes improvements in patients with disuse osteoporosis

BMD, bone mineral density; DEXA, dual-energy X-ray absorptiometry; uTFA, unilateral transfemoral amputation.

**Figure 2.**
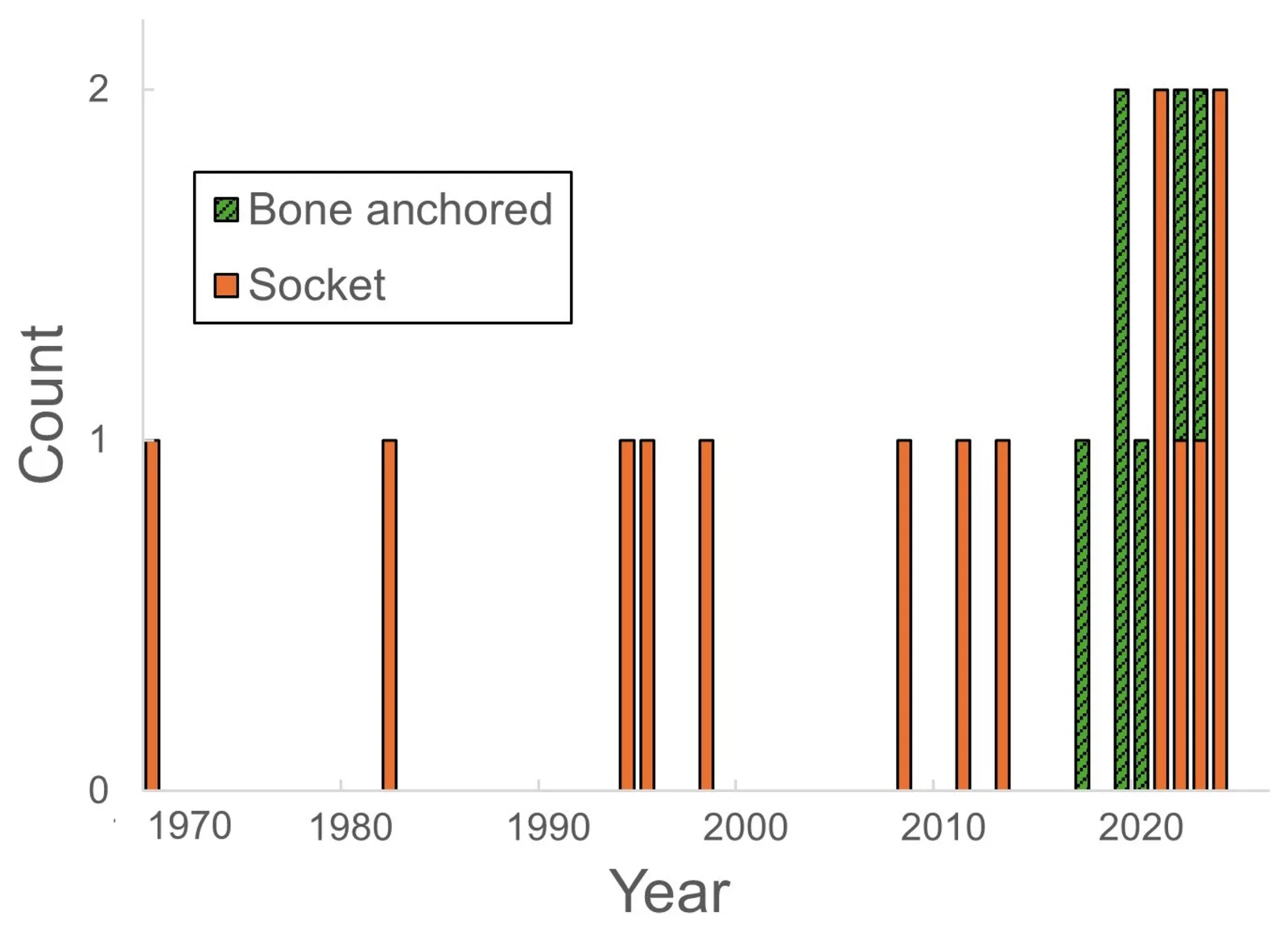
Frequency of published articles by year and by prothesis design.

Included articles were published between 1969 and 2024, with 45% published in the last 5 years, as shown in Figure 2. In the socket prosthesis group, prior to adoption of DEXA as gold standard for BMD measurement in the early 90s,^54^ two articles reported results with X-ray films and used expert opinion to report on bone status.^1,40^ Two articles used CT scans, one used the non-amputated limb as control,^5,43^ while Hoyt et al. developed a customized algorithm to transform CT readings into DEXA presenting results in *T*-Score.^5^ Lastly, one article used MRI readings to measure bone density in the hip of the amputated leg,^41^ as presented in Table 3.

**Table 3.** Utilized imaging techniques and bone density scales in the traditional socket prosthesis group

Bone status grading scale	Imaging technique			
	X-ray	DEXA	MRI	CT
Expert opinion	Sevastikoglou et al.^1^; Benichou & Wirotius^44^			
Comparison with non-amputated limb		Rush et al.^3^	Jaegers et al.^49^	Ramirez et al.^46^
*T*-Score		Kulkarni et al.^4^; Sherk et al.^41^; Cavedon et al.^43^; Hoyt et al.^45^; Jin et al.^47^		Hoyt et al.^5^
*Z*-Score		Kulkarni et al.^4^; Flint et al.^48^; Cavedon et al.^54^; Yasmeen et al.^42^		

CT, computed tomography; DEXA, dual-energy X-ray absorptiometry; MRI, magnetic resonance study.

All articles in the bone-anchored prosthesis group used DEXA for BMD measurement and all articles reported BMD comparison values using ipsilateral limb readings over time.

Factors assessed for association with BMD status as reported by the prosthetic socket suspension literature are presented in Table 4; none were reported in the bone-anchored suspension literature.

**Table 4.** Assessment of associations between risk factors and bone status as proposed in the traditional socket prosthesis literature

Risk factors	Correlation	No correlation
Age at time of study	Rush et al.^3^	Flint et al.^48^; Sherk et al.^41^
Age at time of amputation	Rush et al.^3^; Hoyt et al.^5^	Sherk et al.^41^; Hoyt et al.^45^
Residuum length	Kulkarni et al.^4^; Flint et al.^48^; Hoyt et al.^45^; Jin et al.^47^	Rush et al.^3^
Amputation etiology	Jaegers et al.^49^	Jin et al.^47^

## DISCUSSION

The aim of this scoping review was to summarize the current knowledge on BMD status in persons with TFA, separated by prosthetic socket and bone-anchored suspension and with the intention of summarizing available evidence on potential mechanism for BMD status as reported in the literature. While there were clear differences in BMD status between prosthesis suspension types, there was a lack of consensus in the literature regarding the underlying mechanism influencing bone status as well as the imaging techniques and grading scales used.

### PROSTHETIC SOCKET SUSPENSION: BMD STATUS AND PROPOSED MECHANISM

There is an agreement of the natural history of bone demineralization after a TFA in the socket prosthesis and pre-operative bone-anchored prosthesis cohorts: however, there was no consensus on the cause. All reviewed articles reported a diagnosis of low BMD levels, at the hip joint, in the amputated leg with respect to expert opinion, their contralateral side, mean of the adult population (*T*-score), and/or controls of the same age and sex (*Z*-score).

Across articles, there were inconclusive results regarding correlations between BMD level and age at time of study or amputation, residuum length, and amputation etiology (Table 4). The literature did suggest associations between BMD levels and: time elapsed since amputation,^3,41,42^ first ambulation,^5,44^ first DEXA recording,^44^ and residuum flap coverages.^5^ Finally, there were no reported associations with pain,^1^ residuum length,^41,42^ bisphosphonate and anticoagulants treatment,^44^ calcium intake,^42^ vitamin D, alcohol consumption, smoking history, mobility levels,^42^ and sex.^44^ However, the only study with results regarding sex effects reported status on mainly young individuals (mean age of 24 years),^44^ suggesting that more focalized research is needed.

Reviewed articles revealed that according to the potential causes of BMD loss, rehabilitation has focused on minimizing disuse atrophy,^4,5,40,44,46^ reducing the amount of immobilization during recovery,^5,44,46,47^ improving gait symmetry and overall kinetic exposure during stance phase,^4,5,42,47^ and supporting bone-chemical composition.^44,45,47^ There was recognition of the potentially modified kinetic chain due to prosthesis use^5,6,43,44,46,47^ and its effect on bone mechanical stimuli, summarizing that the effect of the “[prosthetic] socket is not fully understood.”^44^

Research on samples with military affiliation^5,6,24,44,46,55–57^ advocated for a rapid and aggressive mobilization therapy as a BMD loss deterrent, and for its additive effectiveness in pain management.^55^ However, the Symposium “Extremity War Injuries VIII: Sequelae of Combat Injuries”^56^ noted that despite efforts at early weight bearing during rehabilitation, hip BMD levels remained low among military Servicemembers. A study of UK, US, and Canadian Servicemembers injured in Iraq and Afghanistan noted that UK military facilities achieved more rapid mobilization (before wound healing) through pneumatic mobility aids in comparison to US and Canadian practice where wound healing is expected before mobilization.^58^ However, a 2020 report with UK military personnel with TFA showed no positive changes in BMD levels.^6,59^

### BONE-ANCHORED SUSPENSION: BMD STATUS AND PROPOSED MECHANISM

Despite the stress-shielding effect, well known in endoprosthetics, no adverse effects on BMD were reported in the bone-anchored prosthesis studies. Reviewed studies indicate that after the surgical recovery period, BMD levels showed a modest increase with a positive trajectory over time. Articles in the bone-anchored prosthesis group reported BMD benefits as early as 12 months following successful implantation procedures, two with statistically significant increases^18,52^ and four with sustained levels of BMD.^39,50,51,53^

However, it is important to consider the length of the residual femur in conjunction with stress-shielding effect related to the implanted anchoring element. Thomson et al. stated that in a third of their subjects with a short residual limb length, a distal implant was inadequate in providing structural support and that an additional implant at the femoral neck had to be introduced for stabilization.^52^ BMD improvements were exclusively in subjects with long residual limbs that only required the distal anchor. Although a metallic implant in those patients with short residual femurs presents a challenge to measuring hip BMD levels, authors confirmed the low bone density status.

Finally, it was also shown that time post-surgery was positively correlated to BMD recovery,^18^ in contrast to the substantial BMD loss in individuals with socket prosthesis that tends to stabilize to a minimum after 3 years.^5^

### BMD STATUS DUE TO THE MECHANICAL INTENT OF THE PROSTHETIC SOCKET

The positive effects of bone-anchored prosthesis on BMD compared to socket prosthesis highlights the importance of sustaining the natural kinetic chain through the femur, as in individuals with transtibial amputation and knee disarticulation.^3,4,42,44,45^ As individuals with bone-anchored prosthesis have been observed to share common gait loading asymmetries with individuals with socket prosthesis,^60^ this suggests that the use of a prosthetic socket may be the differentiating factor affecting BMD level. For instance, the protective mechanical intent of socket prosthesis, which alters the kinetic chain by redirecting weight load bearing to the ischium, may in fact shield the femur at the cost of bone demineralization. The quadrilateral weight-bearing socket redirects 50% of body weight to the ischial tuberosity, 33% to the gluteus, and 17% to the remaining soft tissue inside the socket,^61,62^ while the ICS was designed to provide body weight support between 50% and 100% by directing ground forces to the ischium.^35^ Accordingly, it is possible that the socket prosthesis suspension might create a force isolation environment for the residual femur in its effort to minimize delivery of potentially insulting forces to the distal femur.^63^

The isolation of mechanical stimulus becomes relevant when considered through the mechanostat theory, which states that bone enters a remodeling phase when a “Minimally Effective Strain” threshold is reached. If appropriate mechanical stimulation is not attained, then osteoclast activity is overstimulated and bone resorption is activated.^64–66^ According to this theory, long bones are mechanically stimulated through two mechanisms: muscle contraction and load bearing.^67^ Since following a TFA, muscles loose up to 70% of their force contribution,^68^ joint forces through the bone are the primary source of stimulation. As it is not possible to directly measure acetabular pressure during gait while wearing an ICS, analytical efforts involving numerical simulation could help investigate if diminished load is experienced with this design, using methods similar to those conducted on able-bodied anatomy.^63,69,70^ These methods could also inform on how differences in prosthetic socket design (e.g., quadrilateral and ICS vs. sub-ischial)^11,71^ and associated clinical variability^35,71^ could deliver varying levels of residuum load. Further research is needed to explore the relationship between the femur’s isolation environment created by a socket prosthesis and the atypical behavior of factors (i.e., sex, age, mobility level) that are known to influence BMD in the general population.

Although the motivation for sub-ischial sockets was to improve user comfort and hip range of motion,^11^ soft tissue suspension (regardless of compression pattern and intermediate components) and non-pelvis interaction could result in the restoration of the natural kinetic chain (skeletal loading) at the hip. According to the mechanostat theory, users of sub-ischial sockets may then experience proximal femoral loading comparable to those with a bone-anchored prosthesis that could help restore and maintain BMD. This potential warrants further investigation to confirm if sub-ischial socket use may facilitate long-term bone health in persons with TFA. However, it is advised that examinations using socket prosthesis adhere to bone status reporting standards, using DEXA imaging, and reporting following *T* and Z Scores. While longitudinal bone status observations present ipsilateral results, as consistently reported by bone-anchored prosthesis articles.

## CONCLUSION

The body of literature on BMD levels in persons with TFA has consistently reported bone demineralization, with several different proposed causes including gait asymmetry and prosthesis contributions to modified skeletal loading that would encourage bone resorption. In contradiction to consistent reports of BMD loss in users of socket prosthesis, more recent studies in individuals with a bone-anchored prosthesis have reported BMD maintenance or gains, suggesting that the prosthetic socket may be an important factor affecting BMD loss in persons with TFA. Consequently, future work on this topic should consider investigations on the influence of the TFA prosthetic socket interactions with the underlying skeleton system to further inform on mechanisms for BMD loss in this patient group and methods to address this issue.

## ACKNOWLEDGMENT

This work was supported by the Mexican Council of Humanities, Science and Technology (CONAHCYT) through funding provided to the primary author.
